# Hidden in Plain Sight: A Case of Arrhythmic Mitral Valve Prolapse Presenting as Cardiac Arrest

**DOI:** 10.7759/cureus.19327

**Published:** 2021-11-07

**Authors:** Yury Malyshev, Miron Borisov, Suvash Shrestha, Adnan Sadiq, Rohan Patel, Felix Yang

**Affiliations:** 1 Cardiology, Maimonides Medical Center, Brooklyn, USA; 2 Internal Medicine, Botkin Hospital, Moscow, RUS

**Keywords:** pickelhaub sign, mitral annulus disjunction, sudden cardiac death, mitral valve prolapse, arrhythmic mitral valve prolapse

## Abstract

Mitral valve prolapse (MVP) is a well-studied, mostly benign, phenomenon; however, arrhythmic mitral valve prolapse (AMVP) is a rare subtype that can precipitate sudden cardiac death (SCD). Herein, we present a case of a young female with sudden cardiac arrest. Extensive multimodality imaging and follow-up helped establish the diagnosis of AMVP.

## Introduction

Although mitral valve prolapse (MVP) is most often a benign condition, in rare instances, it is associated with malignant ventricular arrhythmias (VA) and sudden cardiac death (SCD). The incidence of arrhythmic mitral valve prolapse (AMVP) in the general population is not known, but it is estimated that SCD occurs in 0.2% - 0.4% patients with MVP per year [1]. Among patients with MVP, the presence of certain echocardiographic features is associated with high risk of VA.

We present a case of a young female with no known medical history who had a cardiac arrest and was subsequently found to have AMVP with evidence of mitral annulus disjunction (MAD) and Pickelhaube sign on echocardiogram. However, these features were not evident initially and were found only on long-term follow-up after a multimodality approach. This case highlights the importance of an exhaustive search for the true etiology of SCD.

## Case presentation

**Figure 1:**
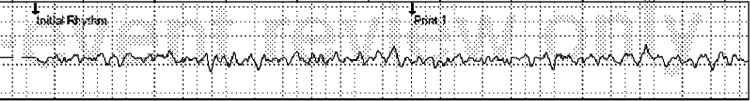
Rhythm strip obtained by emergency medical services on site showing ventricular fibrillation

A 24-year-old lady with no prior medical history was brought to the hospital after a witnessed cardiac arrest. She had no recent illnesses, no family history of SCD, or significant cardiac disease. Patient used marijuana regularly. When emergency medical services arrived at the scene, she was in ventricular fibrillation (Figure 1).

**Figure 2:**
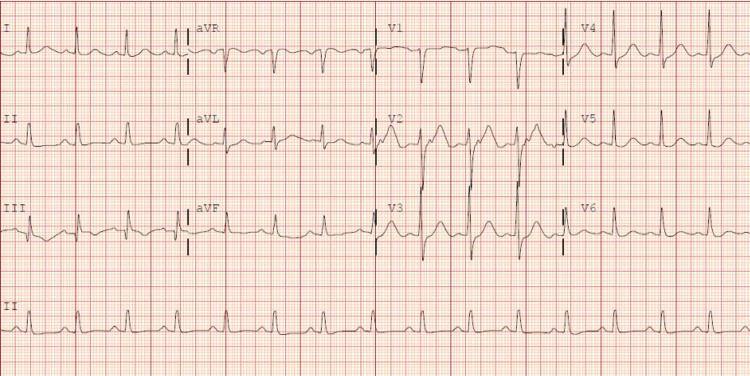
EKG on admission - sinus rhythm, prolonged QTc of 523

She was defibrillated four times and intubated. After 10 minutes, she regained spontaneous circulation. Immediately after arrival at the hospital, the patient was started on hypothermia protocol. Initial blood work revealed hypokalemia (3.2 mmol/l), troponin I peaked at 0.75 ng/ml. EKG showed sinus rhythm, no ischemic changes and QTc of 523 (Figure 2).

However, later QTc improved to normal. Post hypothermia protocol, she had good neurological recovery and was successfully extubated. At this time, we considered the following differential diagnosis: long QT syndrome, ischemia, previously undiagnosed cardiomyopathy. To determine the etiology, the following investigations were done: CTA of the chest which ruled out pulmonary embolism or aortic dissection, and coronary artery CTA which reported normal coronary arteries.

**Figure 3:**
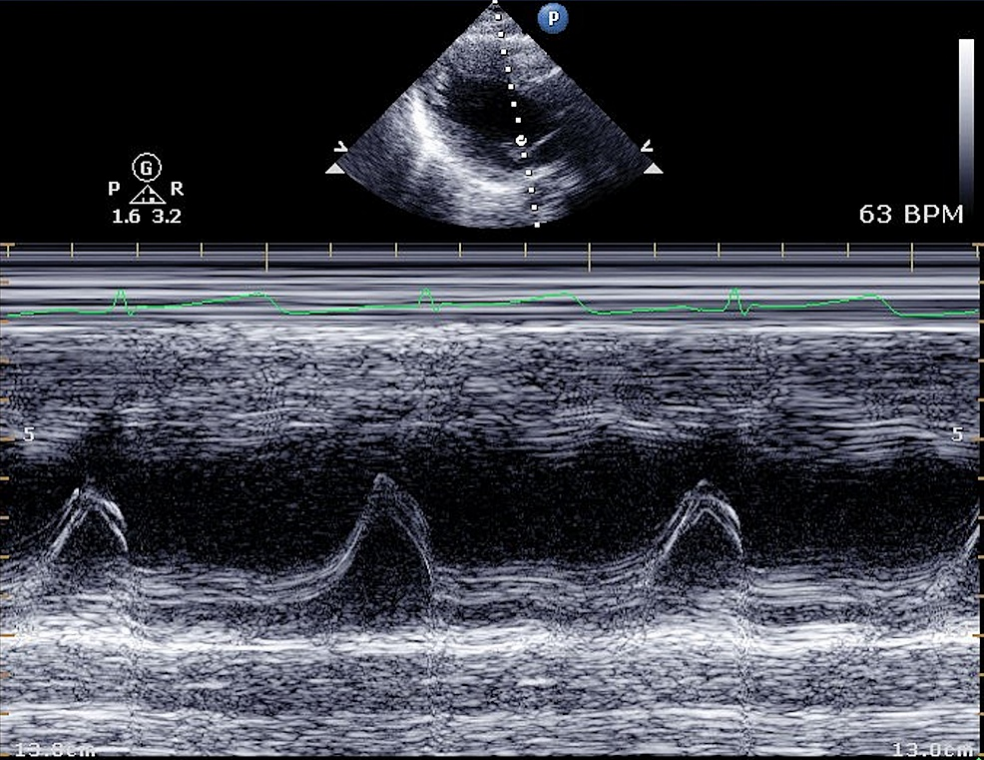
Initial echocardiogram showing M-mode through the mitral valve

**Figure 4:**
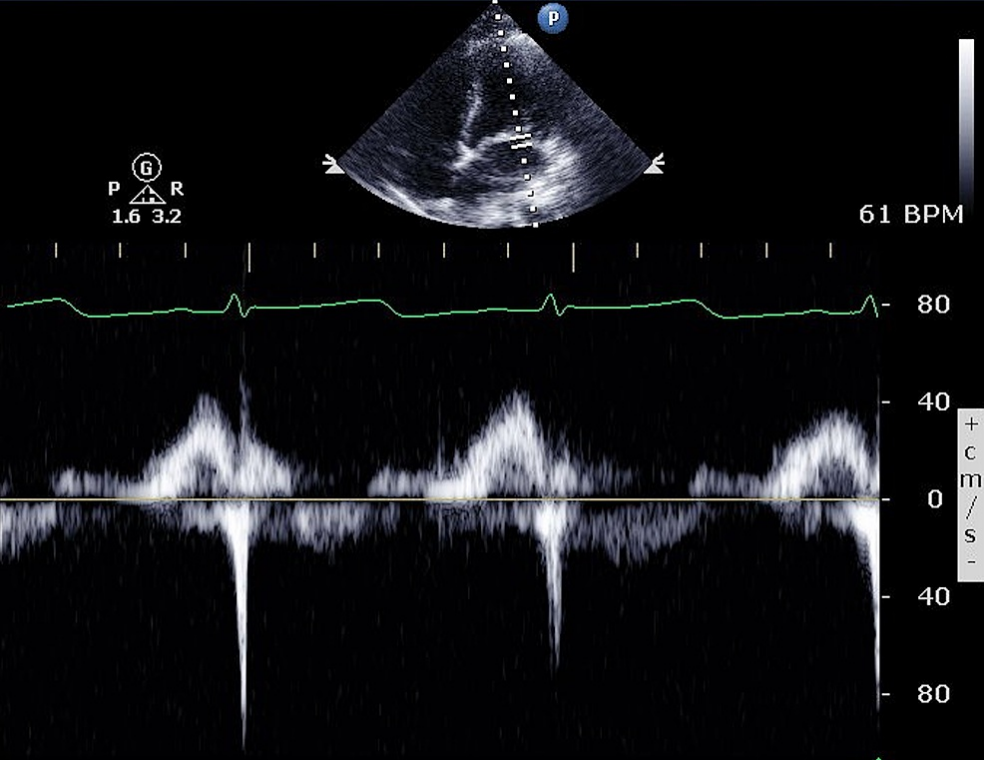
Initial echocardiogram showing pulsed-wave Doppler thought the mitral valve

**Figure 5:**
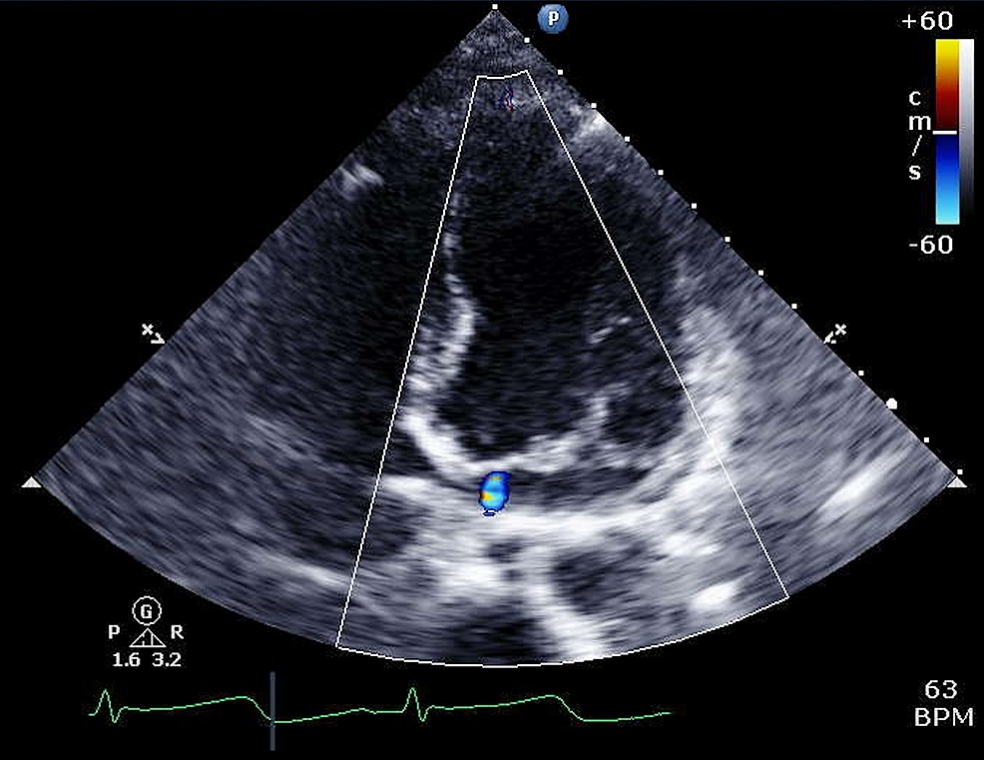
Initial echocardiogram showing four-chamber view with color Doppler

The initial echocardiogram showed an ejection fraction (EF) of 16%-20%, global hypokinesis, moderately reduced right ventricular systolic function, and mild mitral valve (MV) regurgitation (Video 1) (Figures 3-5).

**Video 1:** Initial echocardiogram after cardiac arrest showing dilated and hypokinetic left ventricle without significant mitral valve prolapse or signs of arrhythmic mitral valve prolapse

Repeat echocardiogram during the hospital stay showed improvement in the EF to 51%-55%, with improved right ventricular function, mild thickening and mild holosystolic prolapse of both MV leaflets with mild MV regurgitation (Video 2).

**Video 2:** Echocardiogram prior to discharge from the hospital showing improved left and right ventricular function

**Figure 6:**
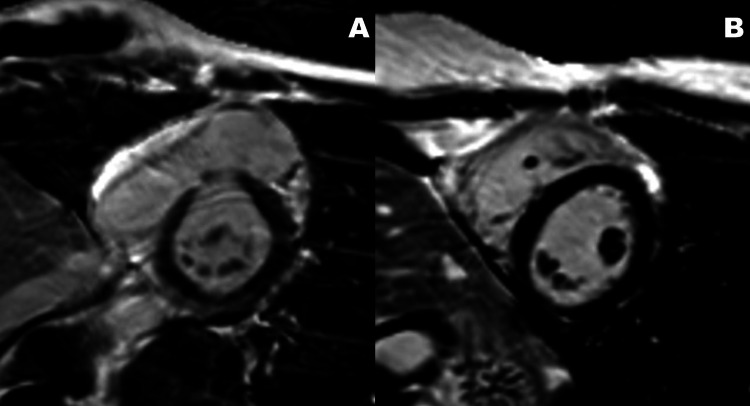
Cardiac MRI with gadolinium A. Short axis at the level of the mitral valve. B. Short axis at the level of papillary muscles.

Cardiac MRI with gadolinium performed one week after the initial echocardiogram showed normal cardiac morphology and function (EF 68%) without evidence of myocardial fibrosis, infiltrative or inflammatory process (Figure 6). 

Patient was initially started on lisinopril and metoprolol for cardiomyopathy, later switched to only nadolol for possible prolonged QT syndrome. A subcutaneous implanted cardiac defibrillator was placed.

Two months after being discharged, she was doing well. A repeat echocardiogram showed normal EF, myxomatous MV, moderate late systolic MVP of both leaflets with moderate MR (Video 3).

**Video 3:** Echocardiogram two months after discharge showing signs of arrhythmic mitral valve prolapse

**Figure 7:**
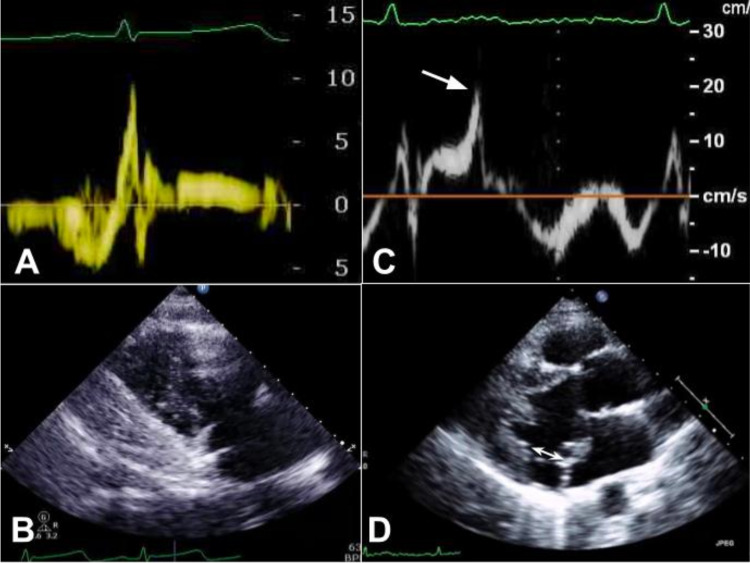
Initial echocardiogram (A, B) and follow up echocardiogram (C, D) A. Tissue Doppler of the mitral annulus without mid-systolic spike. B. Parasternal long axis view, no mitral annular disjunction appreciated. C. Tissue Doppler of the mitral annulus with prominent mid-systolic spike of 20 cm/s - Pickelhaube sign (arrow). D. Parasternal long axis view with significant mitral annular disjunction (double arrow) of 19 mm.

In addition, it showed MAD of 19 mm and Pickelhaube sign on mitral annular tissue Doppler which were not obvious on prior echocardiograms (Figure 7).

## Discussion

MVP is defined as the abnormal movement of any part of the MV >2 mm above the annular plane in parasternal long-axis view on an echocardiogram [2]. It is estimated that 0.2% to 0.4% of patients with MVP per year have SCD. Unfortunately, the exact burden of AMVP is not known and it is likely underdiagnosed [1]. However, it has specific echocardiographic signs, which can also serve as markers for SCD or life-threatening arrhythmias: the Pickelhaube sign and MAD.

MAD is defined as a wide separation between the atrial wall-MV junction and the atrial aspect of the left ventricle free wall [3]. It is diagnosed if the separation is ≥ 5 mm. MAD has profound implications on the dynamic behavior of the MV shape, resulting in paradoxical annular flattening and increased diameter during systole. This alters the physiological saddle-shape of the annulus, which increases stress on the MV leaflets leading to their degeneration suggesting that MAD might be one of the most important precursors of VA [4]. In addition, altered shape of the MV annulus increases myocardial stretch in the inferobasal segments and papillary muscles. The resultant hypercontraction can lead to hypertrophy and replacement-type fibrosis [1]. However, our patient did not have evidence of fibrosis on cardiac MRI. This is not unusual. Observational studies showed that 34% of patients with MAD had VA and only approximately one-third of those had myocardial fibrosis [5]; MAD is associated with a sevenfold increased risk of VA [6]. This supports the idea that MAD itself has a key role in arrhythmogenesis due to mechanical stretch of the myocardium [1]. The severity of MAD correlates with the occurrence of VA. MAD > 8.5 mm is a strong predictor of VA [7]. MAD in our patient was 19 mm.

Pickelhaube sign is a high-velocity (usually > 16 cm/s) mid-systolic spike in the tissue Doppler velocity profile of the MV annulus in patients with bileaflet MVP [8]. The name comes from the German military who wore helmets decorated with a thorn that resembled an echocardiographic spike. It has been suggested that an arrhythmogenic effect arises from the pulling of the posteromedial papillary muscle by the prolapsing leaflets causing the adjacent posterobasal left ventricular wall to be pulled sharply toward the apex [8]. The velocity of the mid-systolic spike in our patient was 20 cm/s. 

Our case also highlights the importance of follow-up imaging in patients with SCD, especially if the etiology is not clear. Dilated left ventricle and hypokinesis after cardiac arrest conceal MVP, because papillary muscles are further away from the MV annulus and pull the leaflets towards the ventricle, preventing the prolapse. Thus, the initial echocardiogram in our case did not show MVP, MAD, or Pickelhaube sign (Figures 7A-7B). As myocardial function recovered, MVP became evident on the echocardiogram and signs of AMVP emerged (Figures 7C-7D). Determining the correct diagnosis can also influence the approach to the screening of family members. 

Unfortunately, specific recommendations for the management of family members of patients with AMVP experiencing SCD have not yet been developed. However, MVP can have hereditary nature. Specific genetic loci that determine familial clustering of MVP have been identified [9]. This raises the possibility of screening unaffected relatives. For more precise screening it might be worthy to consider evaluating patients with “malignant” phenotype: bileaflet MVP, female sex, and frequent complex ventricular ectopic activity [10].

## Conclusions

AMVP is an underdiagnosed condition that can lead to SCD. Echocardiographic signs are identified and should be considered for patients with SCD and MVP. However, as our case demonstrated, AMVP can be hidden in the presence of dilated cardiomyopathy post cardiac arrest. Thus, it is crucial to follow these patients until recovery to establish the proper diagnosis. The question of screening family members of patients with AMVP and SCD is controversial. More studies are needed to establish the correct approach.
